# Correction to: Rapid turnover of life-cycle-related genes in the brown algae

**DOI:** 10.1186/s13059-019-1657-8

**Published:** 2019-02-22

**Authors:** Agnieszka P. Lipinska, Martha L. Serrano-Serrano, Alexandre Cormier, Akira F. Peters, Kazuhiro Kogame, J. Mark Cock, Susana M. Coelho

**Affiliations:** 1 CNRS, Algal Genetics Group, Integrative Biology of Marine Models, Sorbonne Université, UPMC Univ Paris 06, Station Biologique de Roscoff, CS 90074, F-29688 Roscoff, France; 2 Department of Ecology and Evolution, University of Lausanne, 1015 Lausanne, Switzerland; 3 Bezhin Rosko, 29250 Santec, France; 4 Department of Biological Sciences, Faculty of Sciences, Hokkaido University, Sapporo, 060-0810 Japan; 5 Laboratoire Ecologie et Biologie des Interactions, Equipe Ecologie Evolution Symbiose, Université de Poitiers, UMR CNRS, 7267 Poitiers, France

**Correction: Genome Biology (2019) 20:35**

**https://doi.org/10.1186/s13059-019-1630-6**

Following publication of the original article [1], it was noticed that the author names were published with initials instead of full names. The article [1] has been updated.
