# Supplementary material for: Rapid turnover of life-cycle-related genes in the brown algae
Source: Genome Biol. 2019 Feb 14;20:35. doi: 10.1186/s13059-019-1630-6 (PMC6374913; doi:10.1186/s13059-019-1630-6)
Supplement: Supplementary file 2 — Figure S1. Brown algal species used in this study. Figure S2. Visualisation of GO terms associated with generation-biased genes. Figure S3. Structural characteristics of unbiased, gametophyte- and sporophyte-biased genes across brown algal species. Figure S4. Proportions of single copy versus duplicated genes and numbers of orphan genes in the generation biased and unbiased genes. Figure S5. Evolutionary rates for generation-biased genes and sex-biased genes. Figure S6. Non-synonymous substitutions, synonymous substitutions and codon usage bias for unbiased, gametophyte- and sporophyte-biased genes in the four studied species. (ZIP 113664 kb) [file 13059_2019_1630_MOESM2_ESM.zip › Fig.S6.pdf]

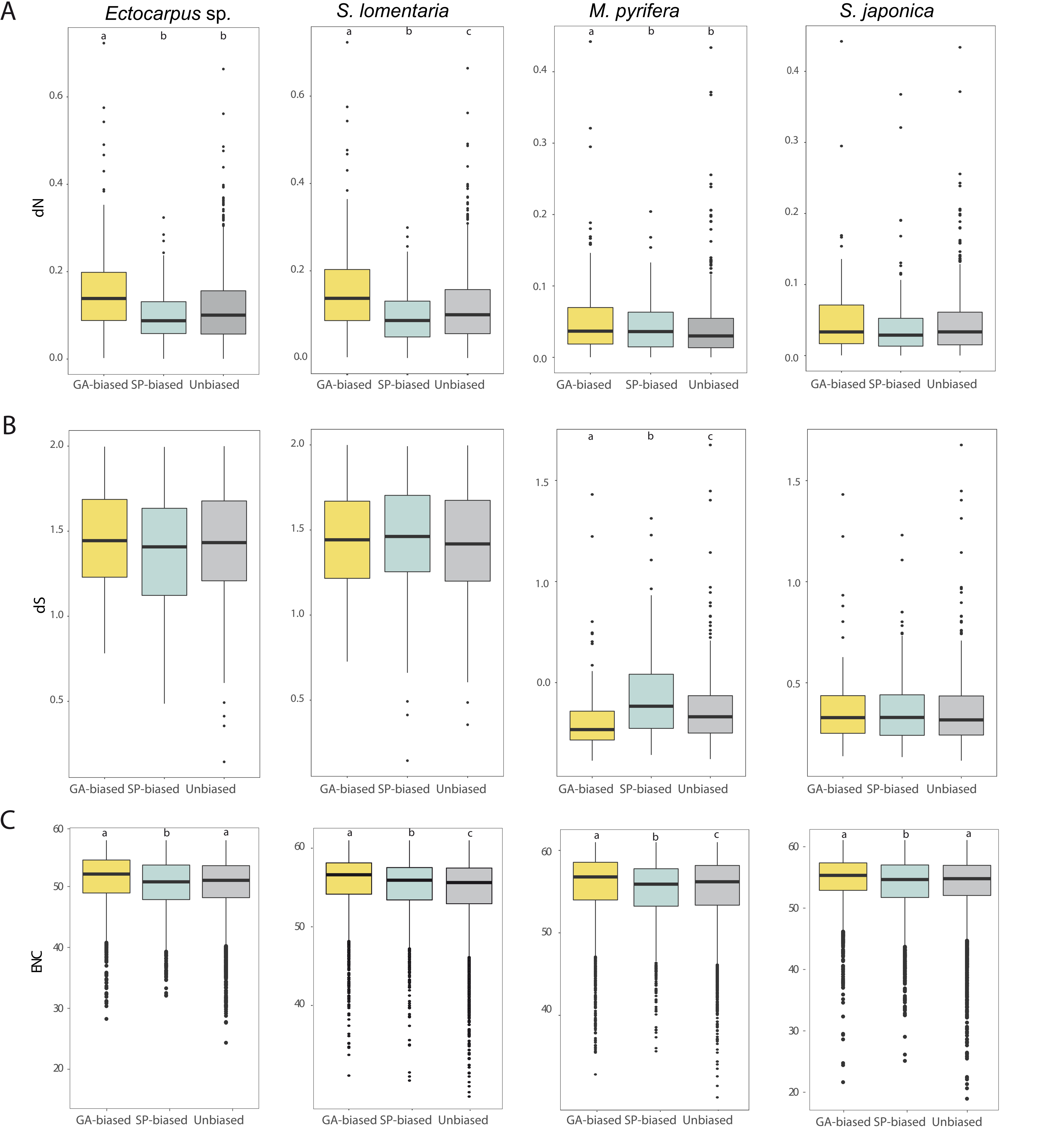

Figure S6. Non-synonymous substitutions (A), synonymous substitutions (B) and codon usage bias (C) for unbiased, gametophyte- and sporophyte-biased genes in the four-studied species. Different letters above the plots indicate significant differences (P<0.05). Statistical significance was calculated for pairwise comparisons using the Wilcoxon rank sum test with Holm adjustment.
